# A simple, fast, and repeatable survey method for underwater visual 3D benthic mapping and monitoring

**DOI:** 10.1002/ece3.2701

**Published:** 2017-02-15

**Authors:** Oscar Pizarro, Ariell Friedman, Mitch Bryson, Stefan B. Williams, Joshua Madin

**Affiliations:** ^1^ Australian Centre for Field RoboticsUniversity of SydneySydneyNSWAustralia; ^2^ Greybits Pty LtdSydneyNSWAustralia; ^3^ Macquarie UniversitySydneyNSWAustralia

**Keywords:** 3D reconstruction, benthic survey, monitoring, mosaic, repeatable survey

## Abstract

Visual 3D reconstruction techniques provide rich ecological and habitat structural information from underwater imagery. However, an unaided swimmer or diver struggles to navigate precisely over larger extents with consistent image overlap needed for visual reconstruction. While underwater robots have demonstrated systematic coverage of areas much larger than the footprint of a single image, access to suitable robotic systems is limited and requires specialized operators. Furthermore, robots are poor at navigating hydrodynamic habitats such as shallow coral reefs. We present a simple approach that constrains the motion of a swimmer using a line unwinding from a fixed central drum. The resulting motion is the involute of a circle, a spiral‐like path with constant spacing between revolutions. We test this survey method at a broad range of habitats and hydrodynamic conditions encircling Lizard Island in the Great Barrier Reef, Australia. The approach generates fast, structured, repeatable, and large‐extent surveys (~110 m^2^ in 15 min) that can be performed with two people and are superior to the commonly used “mow the lawn” method. The amount of image overlap is a design parameter, allowing for surveys that can then be reliably used in an automated processing pipeline to generate 3D reconstructions, orthographically projected mosaics, and structural complexity indices. The individual images or full mosaics can also be labeled for benthic diversity and cover estimates. The survey method we present can serve as a standard approach to repeatedly collecting underwater imagery for high‐resolution 2D mosaics and 3D reconstructions covering spatial extents much larger than a single image footprint without requiring sophisticated robotic systems or lengthy deployment of visual guides. As such, it opens up cost‐effective novel observations to inform studies relating habitat structure to ecological processes and biodiversity at scales and spatial resolutions not readily available previously.

## 1 Introduction

Effective techniques to quantify underwater benthic community composition and physical habitat structure are of importance to marine ecologists and resource managers. Species diversity and abundance data characterize community structure and, when monitored, can be used determine ecological responses to disturbance, ranging from shorter term storms and thermal events (De'ath, Fabricius, Sweatman, & Puotinen, 2012) to longer term stressors associated with climate change (Hughes, 2003). The physical habitat structure built by benthic communities provides diverse niches and supports and array of associated organisms. Benthic habitats with higher levels of structural complexity support greater levels of species abundance and diversity (e.g., fishes and crustaceans) (Graham & Nash, 2013), and show faster rates of recovery following disturbances (Graham, Jennings, MacNeil, Mouillot, & Wilson, 2015). As a consequence, habitat complexity can be used as an indirect indicator of the health and functioning of some ecosystems (e.g., productivity and trophic redundancy). Enabling fast and reliable observation of benthic community composition and structural complexity over large areas, especially during difficult field conditions, will greatly improve tests of ecological theory and the effectiveness of monitoring programs.

Traditional techniques to estimate community composition and habitat structural complexity are labor‐intensive, low‐dimensional, and capture data at small spatial and temporal scales (Friedlander & Parrish, 1998; Loya, 1972; Luckhurst & Luckhurst, 1978). For instance, the line intercept transect records the one‐dimensional length of overlap with different benthic categories (e.g., coral, seaweed, and sand), which is used as an estimate of the two‐dimensional coverage of these categories. Similarly, a common measure of habitat structural complexity (or rugosity) is the ratio between the length of a chain draped over the benthos and the absolute distance between the start and end points. The limited scale of such techniques requires high levels of replication for accurate estimates. Furthermore, transects and chains are usually set randomly, resulting in a loss of spatial relationships.

Recent advances in computer vision have enabled three‐dimensional reconstructions of bathymetry from which benthic categories (Beijbom et al., 2015; Bewley et al., 2012; Shihavuddin, Gracias, Garcia, Gleason, & Gintert, 2013) and multiscale structural complexity (Friedman, Pizarro, Williams, & Johnson‐Roberson, 2012) can be estimated. Several recent studies have used to off‐the‐shelf Structure from Motion (SfM) software such as Photoscan to build 3D models of colonies and broader reef patches, and characterize the quality of these reconstructions (Burns, Delparte, Gates, & Takabayashi, 2015; Figueira et al., 2015; Leon, Roelfsema, Saunders, & Phinn, 2015; Storlazzi, Dartnell, Hatcher, & Gibbs, 2016), establishing confidence in the use of visual reconstructions to address ecological questions (Burns et al., 2016). These techniques rely on combining overlapping images into a composite 3D reconstruction, and while they can scale to areas of tens to thousands of square meters consisting of tens of thousands of images, they need a systematic way of covering the survey site. Otherwise, poor coverage in the form of gaps or holes (missing imagery for parts of the benthos) or in low overlap (low number of views of the same scene point, resulting in low‐precision triangulations and structure estimates) compromise the usefulness of the imagery. Systematic coverage is an ideal task for a properly instrumented underwater robot, which can carry down‐looking cameras and be preprogrammed to follow a survey pattern to collect the desired imagery (Williams et al., 2012). However, the use of robots is still logistically complex, requiring specialized personnel in the field, and robots do not operate well in shallow‐water, high‐energy conditions.

Diver‐held imaging systems can also deliver broad area coverage although the replication of systematic “mow the lawn” patterns requires ropes as visual guides and additional people in the water to handle them (Henderson, Pizarro, Johnson‐Roberson, & Mahon, 2013). Others have relied on swimming an approximate grid pattern unaided by external guides (Burns et al., 2015), but this approach does not scale well to larger areas, tends to break down for narrow line spacing, or in strong swell or currents (Andersen, 1968). It is also a tedious task that depends heavily on the skill of the diver. A simpler approach, the “minute mosaic” (Gintert et al., 2012) uses a rebar pin as a visual reference for a diver to complete three revolutions with increasing radius. As it depends on the diver's assessment of distance to the pin, the areas covered varied from 19 to 44 m^2^. This is also likely to result in variable image overlap between revolutions, affecting the quality of the composite and limiting its value for repeat surveys. Other domains use spirals to survey areas. For example, Archimidean spirals, that resemble involutes of a circle, are used in surface reconstruction in metrology (Wieczorowski, 2001) and in estimating patchy distributions (Kalikhman, 2006) although these uses are concerned with sparse sampling of the area of interest.

We present a simple, repeatable, and low‐cost method to generate systematic surveys for visual three‐dimensional reconstructions of benthic habitats. It removes the need for high‐end navigation and controls and relies instead on constraining motion of a swimmer carrying the imaging equipment. Consistent coverage is attained using a line wound around a fixed drum as a guide. Unwinding the line under natural swimming tension constrains motion to a spiral‐like pattern. The curve traced by the tip of the line (and the imaging package attached to it) corresponds to the involute of a circle, with constant separation distance between revolutions corresponding to the circumference of the drum. The approach we present is practical for full coverage of ~110 m^2^ areas, which corresponds to a radius of ~6 m. Much larger lengths increase the chances of entanglement.

## 2 Materials and Procedures

**Figure 1:**
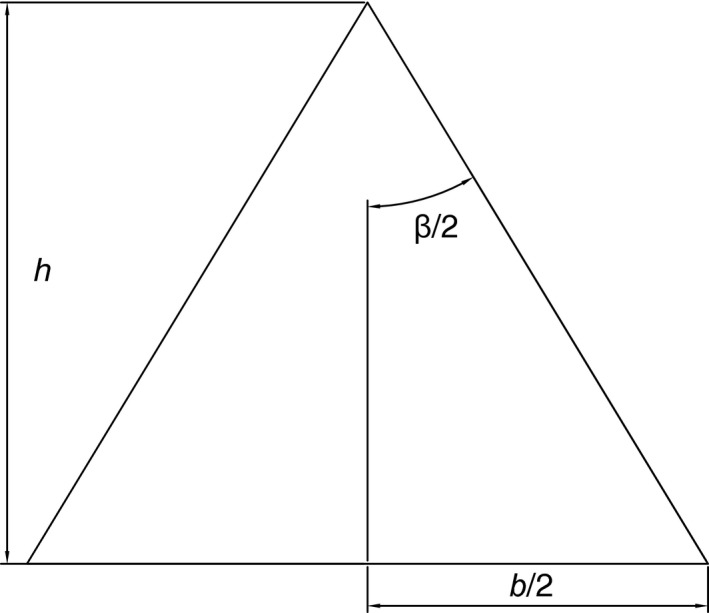
Footprint, *b*, seen by a down‐looking camera with angle of view of *β* at an altitude of *h*. In general, the aspect ratio of imaging sensors is not square so that angle of view across track (across direction of motion) and along track is different. We refer to these by subscripts *β*_across_ and *β*_along_ and likewise for the corresponding footprints *b*_across_ and *b*_along_

**Figure 2:**
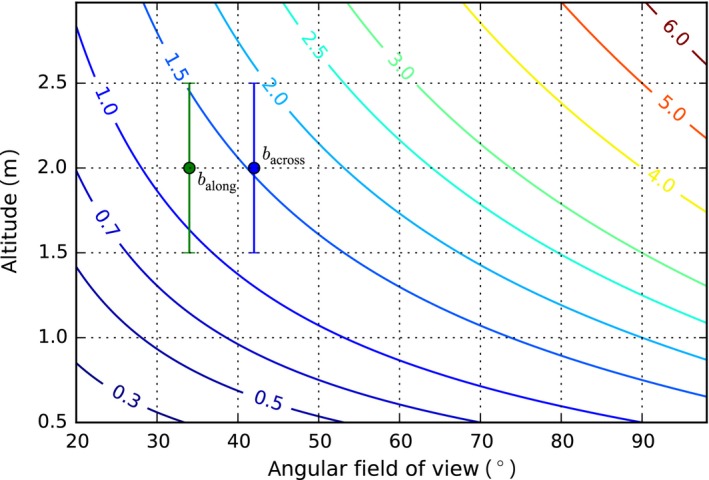
Contours of constant footprint *b* in meters as a function of altitude *h* and angular field of view *β*. Across‐track footprint (blue marker) and along‐track footprint (green marker) for the camera used to generate the results in this study, with a target altitude of 2 m. The effect on footprint of a ±0.5‐m variation in altitude is illustrated by the range bars

For down‐looking cameras, systematic surveys covering areas much larger than the footprint of a single image require multiple views of the same scene points (i.e., “image overlap”) to relate the multiple images into a composite representation such as a 3D reconstruction or an orthographic mosaic. In the case of a down‐looking camera, image overlap along the direction of motion depends on the angular field of view, altitude, and motion between image capture instants (Figure 1). The footprint *b* is given by b=2htan(β/2), where *β* is the angular field of view and *h* the altitude (distance from camera to seafloor). The angular field of view can be estimated from a camera calibration (Bouguet, 2004) or, approximately, using the effective focal length in water and the imaging sensor size. For example, the configuration that captured the imagery used here has an across‐track field of view of 42°, and 34° along track. At a desired altitude of 2 m, the across‐track footprint is *b*_across_ = 1.54 m and the along‐track footprint is *b*_along_ = 1.22 m (Figure 2) giving a footprint of just under 2 m^2^ per image. The footprint size and the displacement between frames Δ determine the number of views of a scene point *n *= *b*/Δ The along‐track displacement is Δ_along_ = *s*·*T*, where *s* is the survey speed and *T* the period between frames. For the purposes of designing a survey with a target number of views, we can determine the displacement as Δ = *b*/*n*. For along‐track motion, the desired survey speed is

(1) $$s=\frac{b_{\mathrm{along}}}{n\cdot T}$$

The trackline spacing (across track) is Δ_across_ = *b*_across_/*n*. For example, for the footprints of our configuration, three views of each scene point (*n* = 3) and a period *T *=* *0.5 s, *s *=* *0.81 m/s, and the trackline spacing should be Δ_across_ = 0.51 m

**Figure 3:**
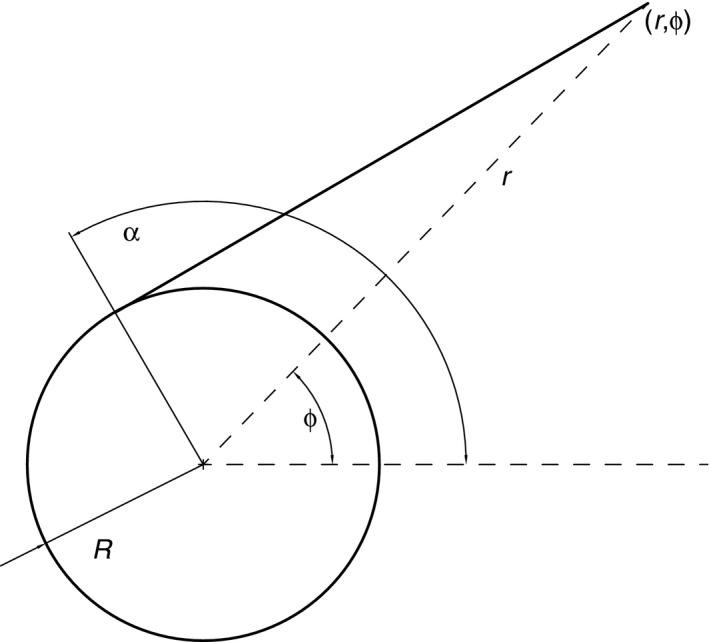
Geometry relating the tip of the unwinding line (*r*,* ϕ*) to the drum diameter *R* and angle along the drum circumference *α*

For a drum (Figure 3) of radius *R* and angle *α* along the circumference of the drum, the tip of the line is located at polar coordinates *r*,* ϕ*:

(2) $$r=R\cdot \sqrt{1+\alpha^{2}}$$

(3) ϕ=α−arctanα

The outer boundary of the involute, *r*, grows in a near‐linear fashion with the number of revolutions, while the path length, *L*, is given by

(4) $$L=\frac{R}{2}\cdot \alpha^{2}$$

growing quadratically with the number of revolutions. From a survey design point of view, the choice of drum diameter and line length, *M*, determines the number of revolutions needed to unwind the line as well as the path length. We assume negligible line thickness in calculating the radius and number of turns around the drum. The survey duration is determined by the swimming speed and path length. The final radius *r*_*M*_ is given by the hypotenuse of the right angle triangle formed by the drum radius and the line extended at right angles, $r_{M}=\sqrt{M^{2}+R^{2}}$, and therefore, the total angle swept to unwind all the line off the drum is

(5) $$\alpha_{M}=\sqrt{{\left(\frac{r_{M}}{R}\right)}^{2}-1}$$

substituting for *r*_*M*_ yields $\alpha_{M}=\frac{M}{R}$ and therefore $L_{M}=\frac{M^{2}}{2R}$. The time to complete a survey, *t*_*M*_, at a swimming speed *s* is then

(6) $$t_{M}=\frac{L_{M}}{s}=\frac{M^{2}}{2R\cdot s}$$

**Figure 4:**
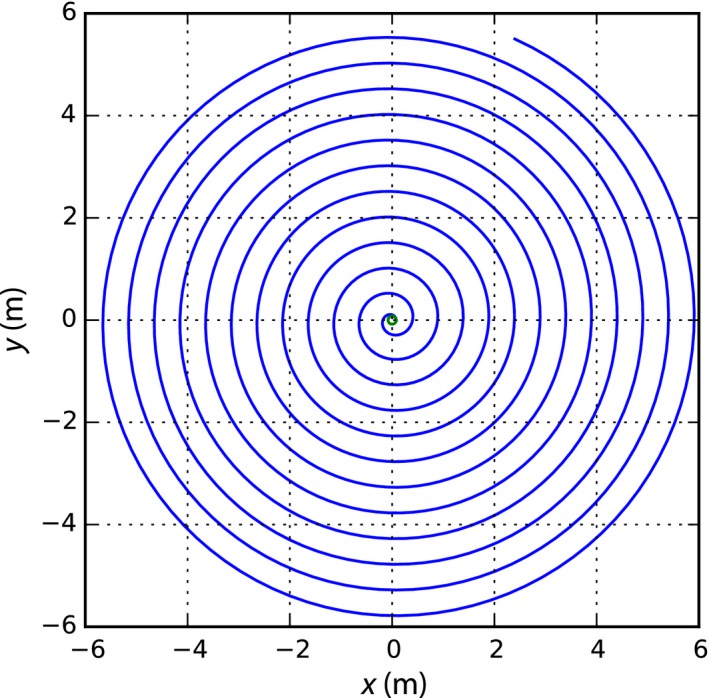
*X*–*Y* trace made by the involute of a circle of diameter 0.16 m and a 6‐m‐long line. In practice, the tip of the line will trace this pattern as it unwound around a drum while keeping it in tension.

**Figure 5:**
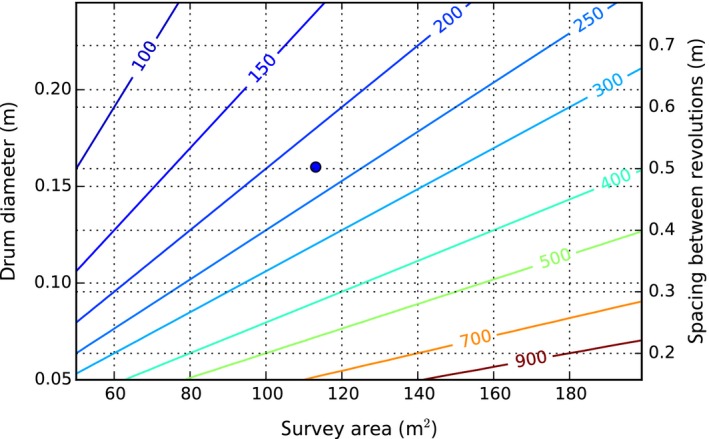
Contours of constant survey path length in meters, as a function of drum diameter (or spacing between revolutions) and desired survey area. The drum diameter sets the spacing between revolutions and should be selected considering the camera footprint (Figure 2) and desired overlap. Given the path length and a swimming speed, the survey time can be calculated. The blue marker indicates the survey design used to generate the results presented in this study, with a drum diameter of 0.16 m and an approximate survey area of 113 m^2^

For a line 6 m long, a drum of 0.16 m in diameter, the path is shown in Figure 4 and the path length of the spiral pattern is 225 m (Figure 5). At a swimming speed of 0.3 m/s, the spiral pattern would take 750 s to execute.

### 2.1 Materials

**Figure 6:**
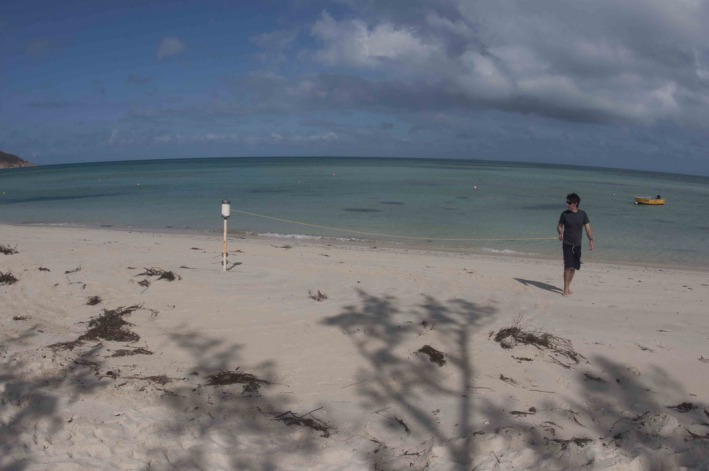
Pole, drum, and line being tested on Lizard Island prior to deployment. Photo by Thomas Bridge

**Figure 7:**
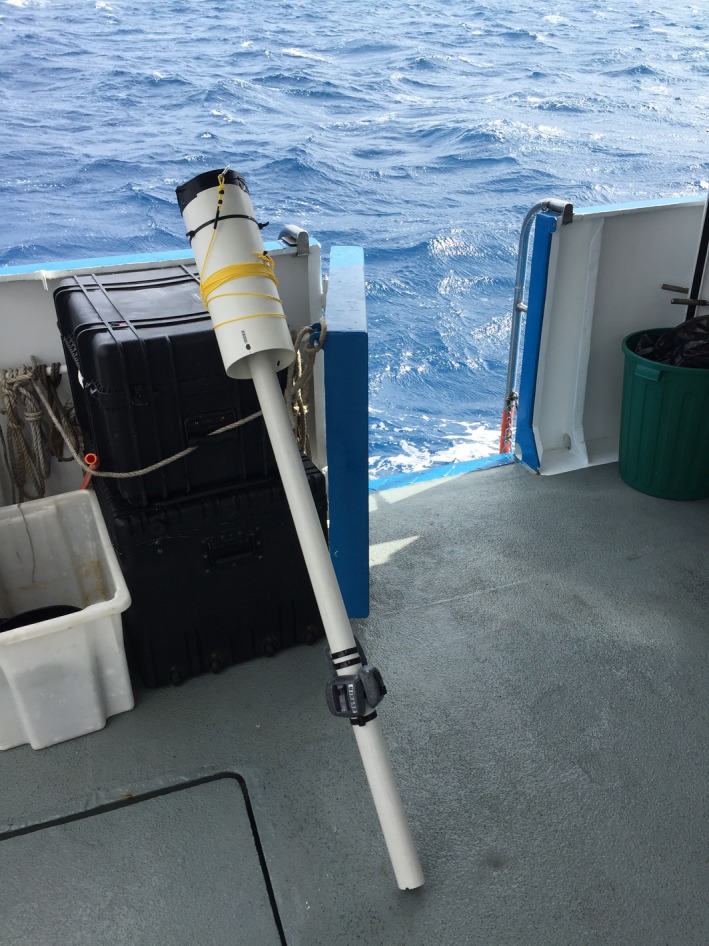
Pole, drum (16 cm diameter), and line

We rely on three‐three major components for data acquisition:

1. *Camera system*. We use a version of the instrumented stereo pair used in Camilli, Pizarro, & Camilli (2007), Henderson et al. (2013), in a smaller form factor and including an acoustic altimeter and a calibrated stereo pair (Johnson‐Roberson, Bryson, Douillard, Pizarro, & Williams, 2013; Mahon, Williams, Pizarro, & Johnson‐Roberson, 2008). A single camera can be used with appropriate external references and processing tools to recover scale, and if necessary, absolute position (see Section 2).
2. *Drum and line*. The diameter of the drum is determined by the desired spacing between successive revolutions. The drum is attached to a pole of length close to the target altitude, typically 1.5–2.0 m. See Figure 6. A simple version can be constructed from PVC pipe and plastic cable ties. The version used in this study has polyurethane sheet rolled in between the PVC pole and drum as a spacer and flotation. We attached dive weights near the base to have a near‐neutral pole that stands upright in water (see Figure 7).
3. *Star picket or base*. For reef structures, a star picket driven into the substrate at the center of the survey patch serves as the anchor point to hold the drum and pole. The pole is keyed to the holes on the star picket so that a pin or screwdriver locks the pole from rotating around the star picket. See Section 2.3 for a discussion on using the method with other bottom types.

### 2.2 Methods

**Figure 8:**
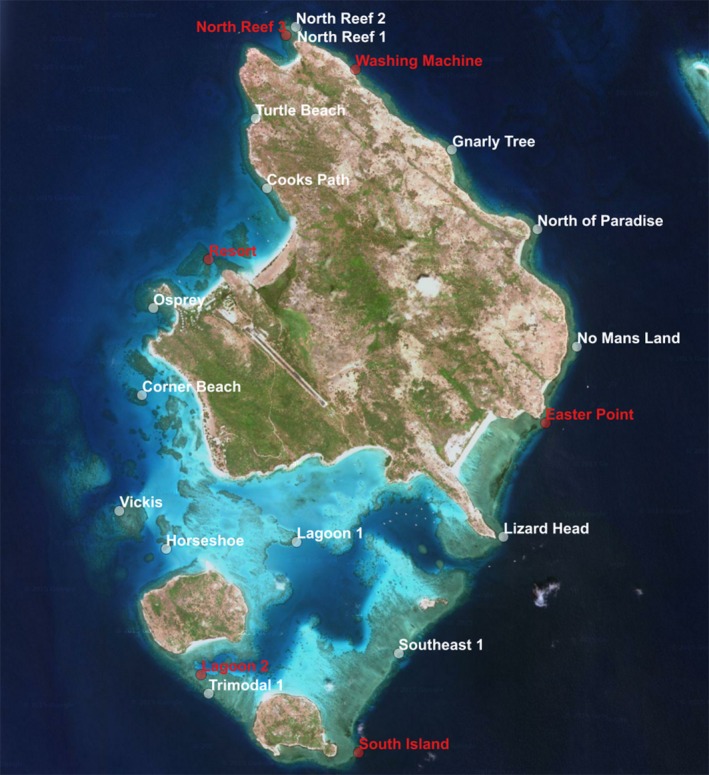
Locations of 21 cyclone recovery spiral surveys around Lizard Island. These sites have been surveyed four times since April 2014. Finding the site to repeat the surveys typically took a few minutes given a GPS coordinate and a mosaic of the previous survey. Red point corresponds with spiral surveys shown in Figures 18 and 19

We characterized the technique's performance based on a cyclone recovery monitoring program involving 21 shallow reef flat (approx. 1–2 m depth) sites around Lizard Island on the Great Barrier Reef (Figure 8). They serve as a time‐series of reef samples of different levels of exposure to cyclones Ita (April 2014) and Nathan (March 2015), as well as prevailing wave forcing from south easterly trade winds. Therefore, the 21 sites capture a broad range of habitats and fieldwork conditions, ranging from sheltered back reef and lagoons through to exposed reef crests.

**Figure 9:**
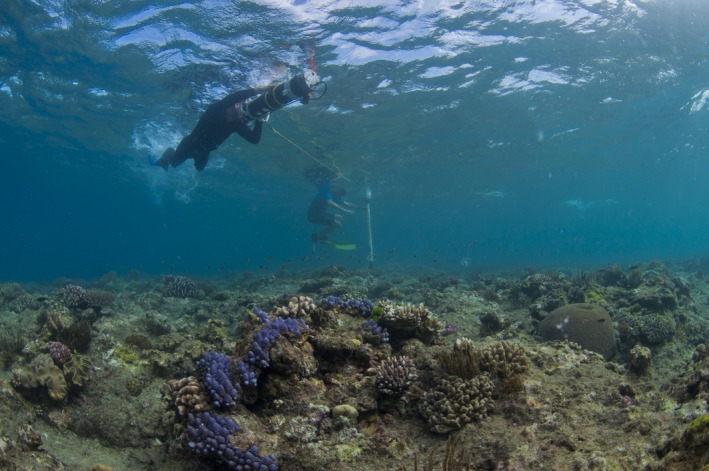
Collecting imagery with a spiral survey in the field. Photo by Thomas Bridge

At each site, the following protocol is followed by two people (a swimmer and an assistant): 1) Select location, such that the spiral covers the area of interest. For site revisits, relocate site with GPS coordinates and printout of previous trip's mosaic. 2) Drive star picket at the center of this area. For site revisits, the picket hole from previous fieldtrip was typically visible. When not, the printout is used to identify the central area of the survey and the position for picket. 3) Attach pole and drum to star picket. 4) Clip imaging package to line. 5) The swimmer then pushes the imaging package forward while keeping line tension and the desired altitude. Continue until the line has completely unwound from the drum. The assistant stays by the pole tending the line (Figure 9). 6) Once the survey is completed, the swimmer detaches the line from the imaging package. The assistant coils the line and takes the pole and drum off the star picket. 7) Optional: the swimmer goes over the center of the survey, completing a two to four passes over the star picket. The primary purpose of these is to have additional coverage of the center of the survey. In cases where overlap between revolutions is small or significant wave disturbances, these extra images can help constrain the reconstruction by providing some imagery across the spiral path. Performed after the main survey, these “spokes” take 1–2 min and are easy to leave out of any further processing if desired. 8) Pick up the star picket.

This procedure produces overlapping images both along and across track, enabling processing imagery into visual 3D reconstructions witouth gaps in coverage, The image‐based reconstruction will not be georeferenced unless additional external references are used. For completeness, we mention some approaches to georeferencing these reconstructions. In the case of our pose‐instrumented package, we also have a noisy observations of GPS (if on the surface), depth and heading, pitch, and roll while performing the spiral survey. The data are processed using the ACFR pipeline (Johnson‐Roberson, Pizarro, Williams, & Mahon, 2010; Johnson‐Roberson et al., 2013; Mahon et al., 2008) to estimate camera poses and 3D composite meshes of the surveyed area. Multiscale structural complexity indices such as rugosity are then derived from those meshes (Friedman et al., 2012). For a single camera setup, SfM packages require additional steps to recover scale and georeferenced position and orientation (e.g., (*x*,* y*,* z*) position of at least two points and the depth *z* of a third point on the survey area). This can be performed, for example, by surveying in points in the survey or artificial markers using surface GPS (Burns et al., 2015) or in shallow‐water near‐shore cases, using a total station theodolite approach (Henderson et al., 2013).

### 2.3 Survey consistency

To quantify the image overlap consistency of the spiral survey procedure, we also performed six surveys using the “mow the lawn” method (Henderson et al., 2013; Mahon et al., 2011). Note that these surveys were conducted on sheltered reef and required three swimmers.

**Figure 10:**
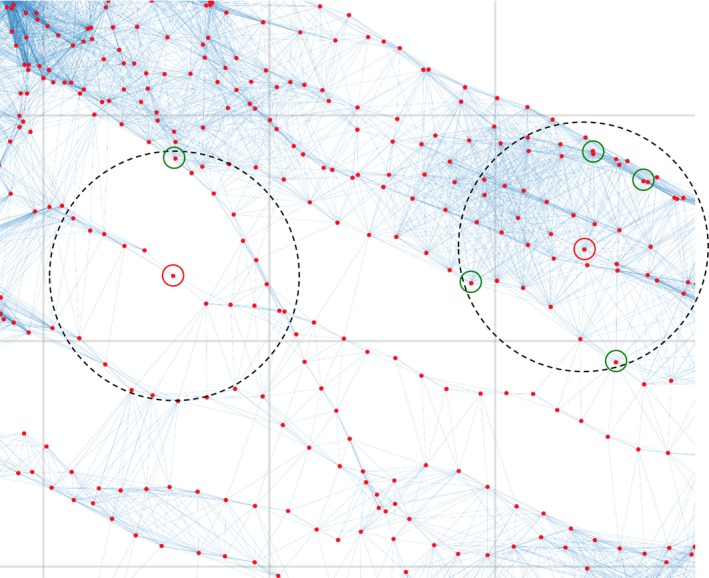
Illustration of a poorly constrained group of cameras (left side dashed circle) and a well‐constrained group (right side dashed circle). The path length in links between the central camera (red circle) and a example cameras (green circle) is short for a well‐connected network. Long distances correspond to “holes” in the network

Any survey pattern should ensure image overlap across track, with each image observing common scene points with other nearby images, and thus forming a well‐constrained photogrammetric network that produces reliable estimates of camera poses and 3D scene points. We quantify this effect by comparing the local density of connections between cameras in the photogrammetric networks formed by the “mow the lawn” and spiral patterns. Specifically, we propose a metric based on the shortest path along linked images in the resulting photogrammetric network. For example, if images are directly linked to each other, the shortest path length between them is one; if they have to go through another image, the shortest path length is two. A “hole” (lack of matches because of poor or nonexisting overlap) between two spatially close cameras requires a long path through several other cameras. We considered six “mow the lawn” and 33 spiral surveys. For each one, we calculated the median length of direct links between cameras and then define a circular neighborhood using a radius twice that size to consider cameras that are “close.” This provides invariance to the size of the image footprint (which changes with imaging altitude). For each camera in a survey, we find the shortest path (in number of links) to all nearby cameras within this neighborhood. Figure 10 illustrates the metric.

## 3 Results

### 3.1 Operational simplicity and survey speed

The equipment used is easy to handle. Driving a star picket temporarily into the reef is a standard task for field ecologists. Once clipped onto the line, the swimmer only needs to advance while keeping tension on the line and maintain a desired altitude. The time to perform a spiral survey is consistent, with variations depending on currents and waves. During the May 2015 field season, we performed 35 spiral surveys comprising the 21 monitoring sites and additional plots for testing repeatability of estimates under varying conditions. The average duration was 15:26 (*SD*: 01:43), with a maximum of 18:47 and a minimum of 12:58. See Table 1 for details. In the case of snorkeling on reef flats, tides affect water depth which determines the imaging altitude and image footprint (see Section 2 and Figure 1). Swell and currents act as disturbances that affect speed and the actual path followed (the line only constrains motion away from the pole). In comparison with “mowing the lawn” (Mahon et al., 2011), our approach is significantly simpler and more reliable. Table 2 contrasts these two survey techniques.

**Table 1:** Survey statistics for the May 2015 field campaign on Lizard Island

	Avg	*SD*	Max	Min
Duration	15:26	01:43	18:47	12:58
Stereo pairs	1,853	205.3	2,255	1,557
Image matches	19,286.3	7,098.1	33,168	6,194

**Table 2:** Qualitative comparison between a “mow the lawn” and spiral surveys for contiguously covering an area with overlapping imagery

Parameter	“Mow the lawn” survey	Spiral survey
Personnel	**Three**. One swimmer and two asssistants managing the guide line	**Two.** One swimmer and an assistant managing the pole and drum
Equipment	Two parallel lines fixed to substrate marking the start and end of each parallel leg, one guide line held by assistants and followed by swimmer. Stakes or similar to fix parallel lines	Pole with drum and line. Star picket and hammer
Preparation effort	**High.** Lay visual guides for two parallel sides of box (10–30 min)	**Low.** Drive star picket and fasten pole (1–2 min) Drive star picket and fasten pole (1–2 min)
Skill level of swinmmer	**High.** Keep constant distance to guide line while swimming in a straight line, turn 180° at end of each line	**Low.** Swim forward and keep tension. Unclip camera from line once fully uncoiled
Skill level of assistants	**High.** Maintain constant step size between tracklines, coordinate with assistant at other end of guide line to move same amount when swimmer completes a trackline, keep guideline over same place in the presence of currents and waves	**Low.** Keep pole roughly vertical. Remove pole once line is uncoiled from drum and exit survey area with pole and line
Reliability	**Low.** Difficult to achieve tight trackline spacing. Prone to gaps across parallel tracklines, loss of synch between assistants resulting in lines that are no longer parallel	**High.** Robust to swell and currents. Easy to achieve tight trackline spacing using narrower drum
Survey area and size	Length of the legs limited to visibility for ease of communication between assistants. 150+ m^2^ possible	radius limited to visibility for coordination between swimmer and assistant. 150 m^2^ possible
Revisit effort	**High.** If lines have to be laid out again	**Low.** Find center of survey and repeat standard preparation

### 3.2 Survey consistency

**Figure 11:**
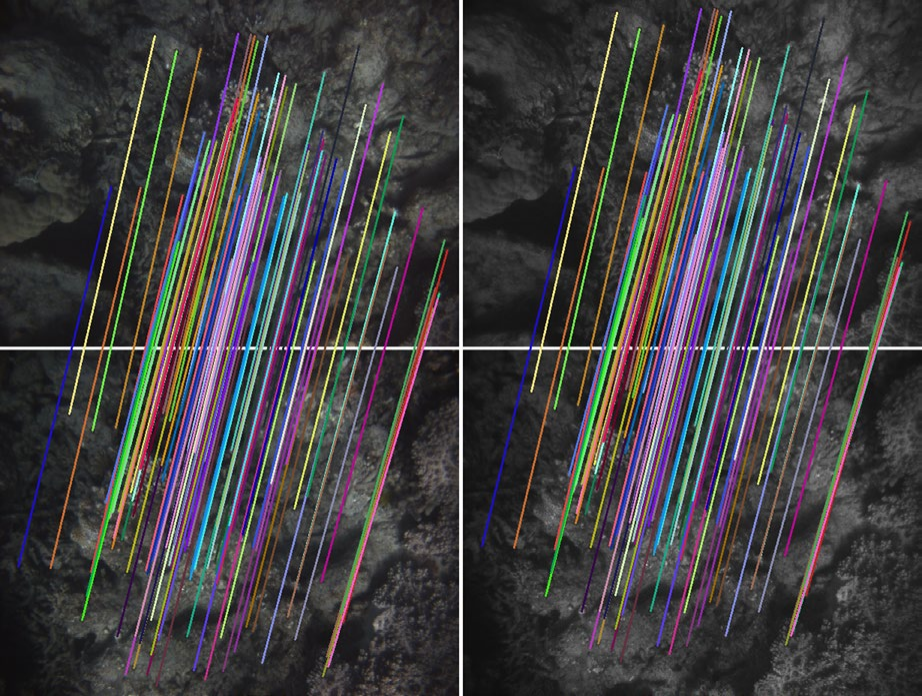
Feature matches between consecutive stereo pairs (top row and bottom row). The colored lines' start and end points correspond to the same feature on the first pair and second pair

**Figure 12:**
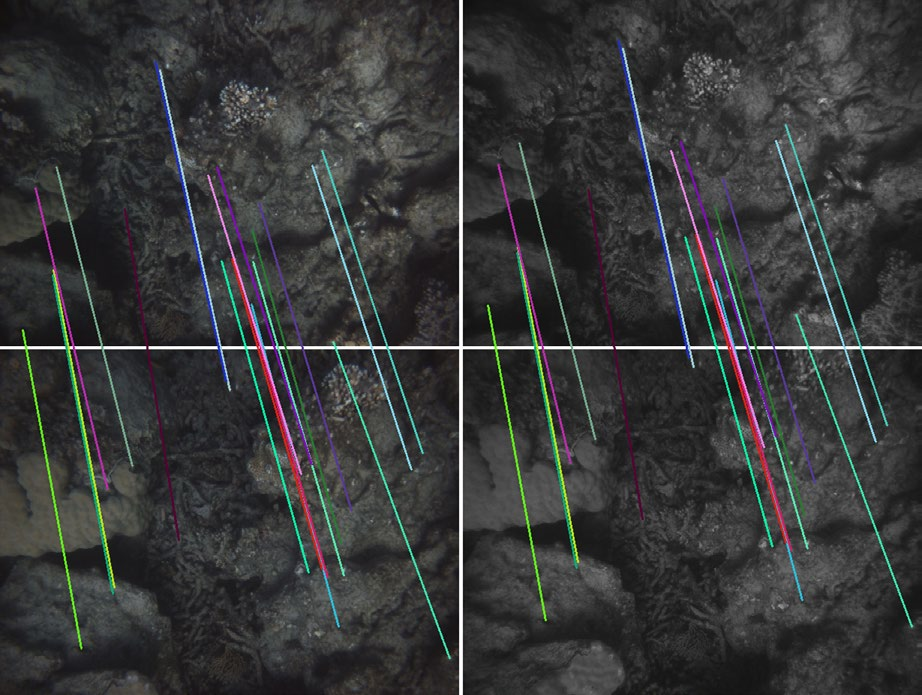
Feature matches between two stereo pairs (top row and bottom row) across two revolutions of the spiral pattern. The colored lines' start and end points correspond to the same feature on the first pair and second pair. The reduced overlap results in fewer matches when compared to Figure 11

The spiral survey by design results in constant separation between revolutions. When matched to the field of view and altitude, it guarantees high overlap. Image features are matched automatically between image pairs to provide estimates of the relative pose of camera positions both across and along track. Figures 11 and 12 show examples of along‐ and across‐track matches between pairs of images.

**Figure 13:**
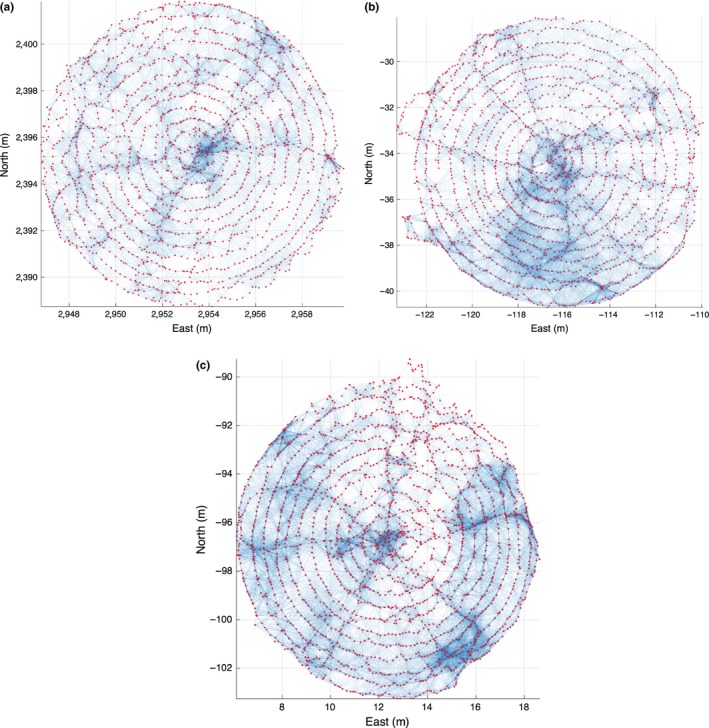
Three examples of spiral surveys. *X*–*Y* plan view, with red dots marking estimated camera positions, and blue lines indicating overlap between camera poses

For the results in this study, we acquire stereo pairs at 2 Hz, providing ample overlap along track. Figure 13 shows examples of spiral surveys. The black dots represent the estimate location of the camera throughout the survey. The red lines join camera locations for which image features have been matched, representing effective image overlap. The green line represents the GPS fixes collected throughout the survey.

**Figure 14:**
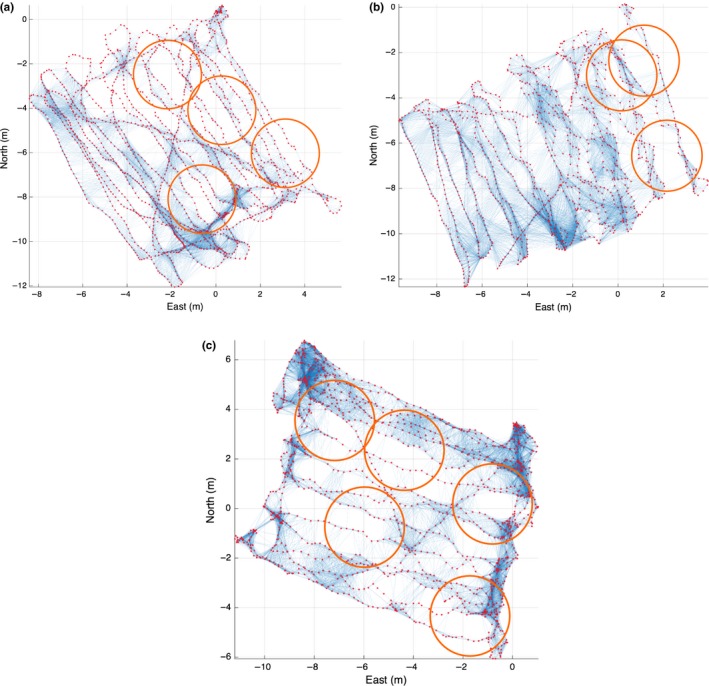
Examples of “mow the lawn” patterns. *X*–*Y* plan view, with red dots marking estimated camera positions, and blue lines indicating overlap between camera poses. Orange circles indicate areas with holes in cover (insufficient overlap)

Figure 14 shows “mow the lawn” patterns where swimmers failed to move the camera properly, resulting in large holes or gaps.

**Figure 15:**
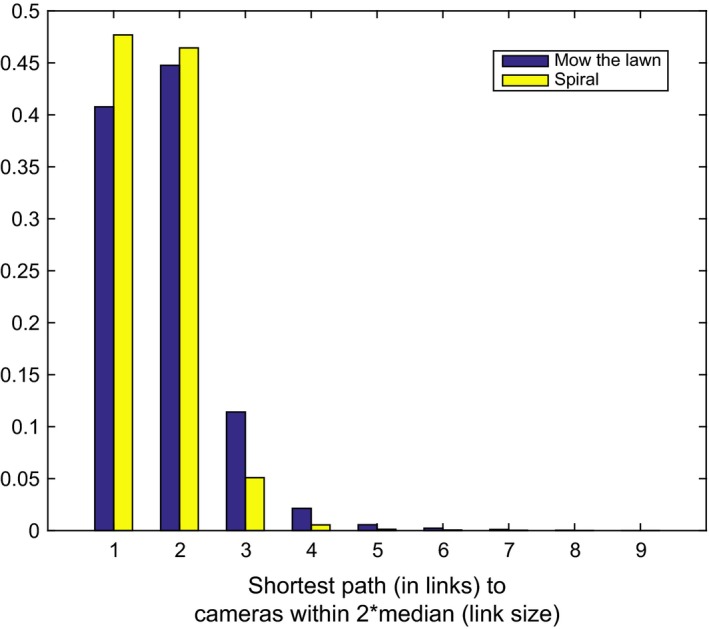
Normalized histogram of the minimum distance or path length (in links) between camera poses within a radius of twice the median length of the links in a survey, based on six “mow the lawn” surveys and 33 spiral surveys in similar conditions and terrain. The minimum distances distribution for the “mow the lawn” surveys has a longer tail and less mass in the one and two link distance bins. Greater minimum distances correspond to holes in coverage or images that do not overlap enough to reliably find common features between them, leading to poorly constrained photogrammetric networks (see Figure 10)

The distribution of the lengths of the shortest paths is indicative of the quality of the survey. Figure 15 shows the histogram of the lengths of the shortest paths to the neighbors of each camera on all the dives. Ideally, the mass of the distribution will be concentrated in shortest path lengths of one and two links. It is clear that the spiral survey approximates this while the “mow the lawn” is skewed to much higher path lengths of three up to nine, indicating the presence of significant holes.

**Figure 16:**
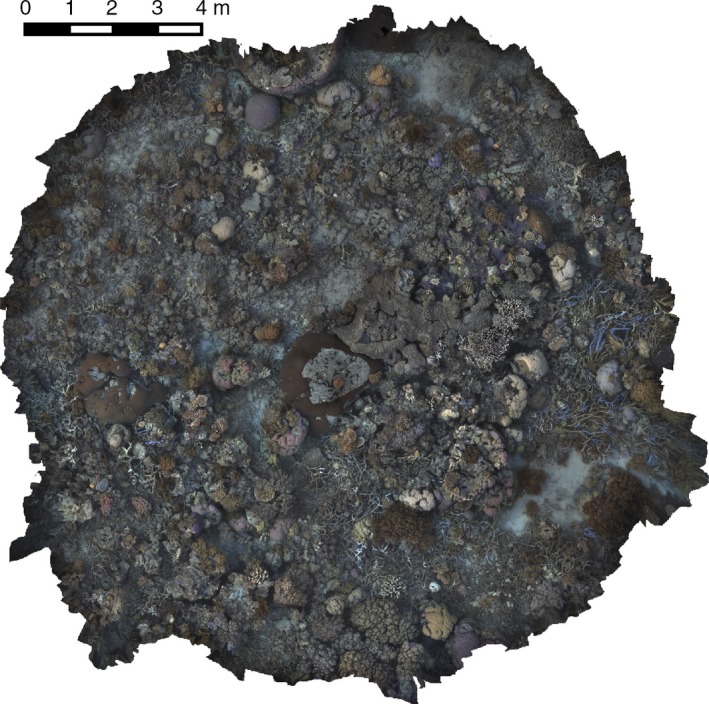
Color photomosaic of a section of Horseshoe Reef, Lizard Island, GBR, reconstructed using postprocessing of approximately 1,600 stereo images

**Figure 17:**
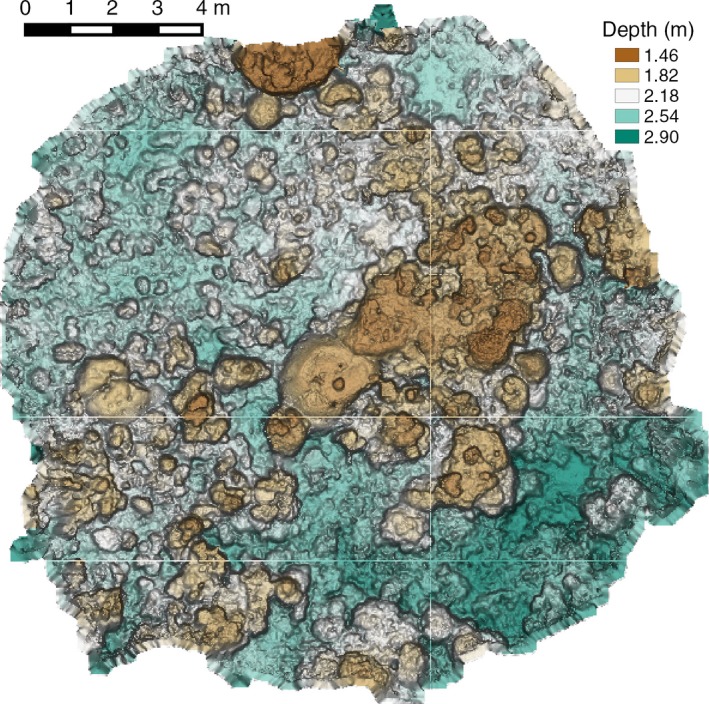
Corresponding bathymetry for a section of Horseshoe Reef (see Figure 16), Lizard Island, GBR

Figure 16 shows an example of the texture‐mapped model for one of the spiral surveys while Figure 17 shows the underlying three‐dimensional surface model.

### 3.3 Revisiting sites

**Figure 18:**
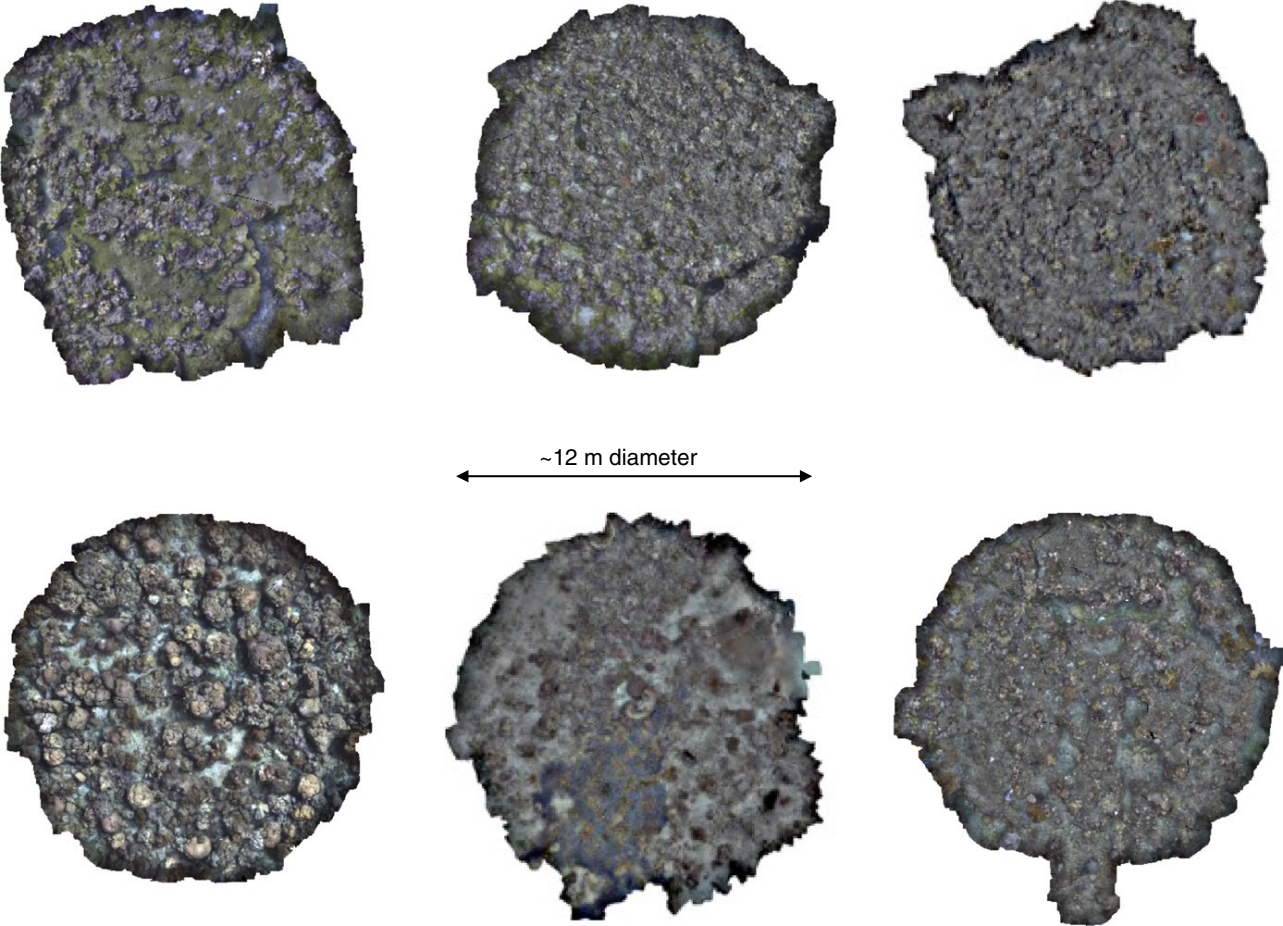
Six spiral surveys collected at sites around Lizard Island showing the variability in the reef cover. Each spiral survey covers an area of approximately 113 m^2^. These are rendered as the orthographic projection of the image‐textured mosaics. The variability in cover is readily apparent. Clockwise, from top left: North Reef 3, Washing Machine, Easter Point, South Island, Lagoon 2, and Resort. See Figure 19 for the corresponding underlying bathymetry and Figure 8 for location of these sites around the island (red points)

**Figure 19:**
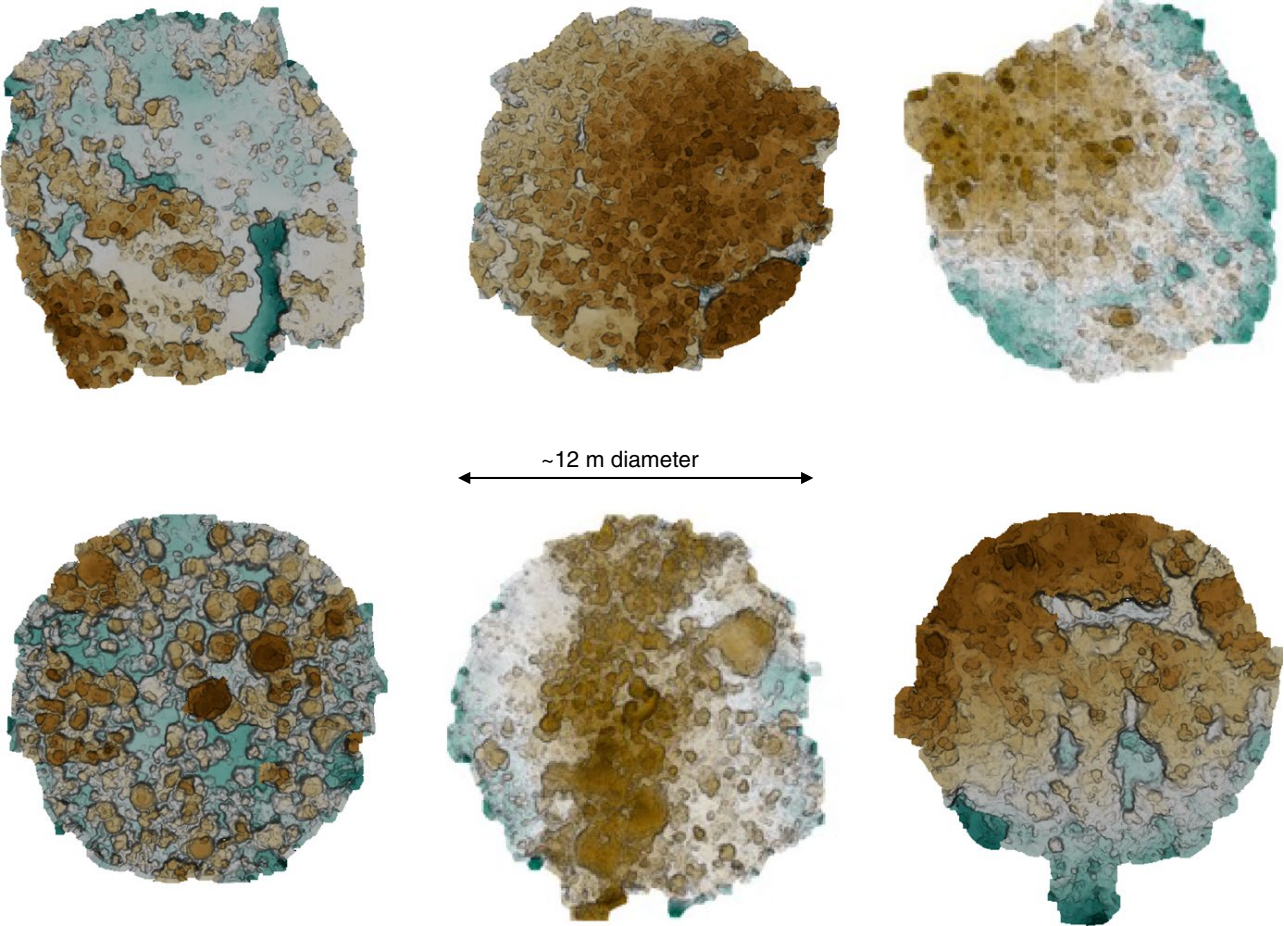
Six spiral surveys collected at sites around Lizard Island showing the variability in the reef cover. Each spiral survey covers an area of approximately 13 m^2^. These are rendered as the orthographic projection of the image‐textured mosaics. The variability in cover is readily apparent. Clockwise, from top left: North Reef 3, Washing Machine, Easter Point, South Island, Lagoon 2, and Resort. See Figure 18 for the corresponding image‐textured mosaics and Figure 8 for location of these sites around the island (red points)

Given a waterproof printout of the mosaic from a previous survey and its coordinates, an experienced swimmer aided by GPS can relocate the central point in seconds to a few minutes, depending on how much the site has changed. We have successfully completed at least 63 revisits of monitoring sites using this approach (21 sites revisited three times, approximately every 6 months) in an area that was subject to a cyclone after the second visit. If the particular application allows the star picket to be left embedded in the substrate, relocating the survey site is trivial. This approach will be robust to substantial changes in appearance that can occur after events such as large storms. Figure 8 shows locations of sites revisited on Lizard Island for April 2014, October 2014, May 2015, and November 2015. Figures 18 and 19 show details of six sites around the island. Our method was able to consistently survey and revisit sites with varying levels of exposure to waves, wind, and currents, ranging from sites in the protected lagoon to those open to ocean swell.

## 4 Discussion

Our constrained motion survey provides a simple yet robust and effective way to systematically cover an area much larger than a single image footprint. The successive passes in the spiral path can be spaced precisely to allow overlap across revolutions and enable 3D visual reconstructions. This approach facilitates georeferencing and revisiting sites for monitoring. With this type of survey data, it is straightforward to generate multiscale terrain complexity measures (Friedman et al., 2012).

This method enables scientists to reliably generate high‐resolution, broad‐scale representations of reef environments without depending on engineering specialists and complex robotic systems. It can be integrated into their standard fieldwork with modest additional effort providing novel views of structural complexity and larger scale spatial patterns. For example, reconstructions from spiral surveys have been color‐printed onto underwater paper and uploaded into underwater tablet GIS software, for in situ coral species identification and habitat feature annotation. When coupled with ecological surveys (e.g., corals and fish), the method can offer valuable data at multiple scales for understanding the relationship between species diversity and habitat complexity. When repeated and coupled with environmental data and observations of the physical disturbances, it enables powerful insights into the ecological and evolutionary processes operating in marine systems.

## 5 Comments and Recommendations

One of the limitations of the technique is that the line between the imaging platform and drum must be free to “sweep” the site unobstructed. This is satisfied by a relatively planar, though not necessarily horizontal, surfaces. It also is satisfied if the center of the survey is at a local minimum or maximum. In practice, constant survey altitude is not achieved and the range of altitude variations encountered by the imaging system needs to remain in focus and provide an image footprint that still achieves overlap with neighboring revolutions at the low end of the altitude range. In cases of significant surfaces that are not captured by a down‐looking camera, it should be possible to complement the systematic spiral survey with additional imagery at oblique angles, as long as there is a sequence of images that gradually change the orientation of the camera while observing the same scene points. This ensures that the additional images can be used by the reconstruction pipeline.

This technique has been mostly used on carbonate reefs, where a temporary or permanent star picket can be driven into the substrate and then serve as an attachment point for the pole. In cases of rocky reefs or soft sediments, different attachment methods are required. An alternative would be to use a pole with a heavy base or tripod. The increase in versatility of bottom types on which the technique comes at the price of a more awkward transport in water. While the results presented in this study are based on surveys using snorkel, it has been used with scuba to collect data at greater depths. In such cases, care must be taken to keep the line length (i.e., maximum radius) under the maximum allowed safe separation distance between divers. In cases of near‐vertical slopes, consideration of the dive profile would also be necessary as the final revolutions would result in changes in depth for the diver comparable to the diameter of the survey.

## Conflict of interest

None declared.
